# Effects of multicomponent exercise intervention on cardiometabolic risk factors in children and young adults with cerebral palsy: a multiple-baseline trial

**DOI:** 10.1186/s13102-024-01006-0

**Published:** 2024-10-21

**Authors:** Tiina Savikangas, Pedro Valadão, Eero A. Haapala, Taija Finni

**Affiliations:** 1 Faculty of Sport and Health Sciences, University of Jyväskylä, PO Box 35, Jyväskylä, FI-40014 Finland; 2 Neuromuscular Research Center, Faculty of Sport and Health Sciences, University of Jyväskylä, Jyväskylä, Finland; 3 Institute of Biomedicine, School of Medicine, University of Eastern Finland, Kuopio, Finland

**Keywords:** Cerebral palsy, Exercise, Strength training, Gait training, Cardiometabolic health, Body composition, Blood pressure, Lipids, Glucose

## Abstract

**Background:**

Adults with cerebral palsy (CP) have a high risk of cardiometabolic diseases. It is unknown whether this risk is elevated in young people with CP and whether exercise can reduce this risk. Therefore, we investigated the effects of the EXErcise for Cerebral Palsy (EXECP) intervention on cardiometabolic risk in children and young adults with CP and compared this risk to typically developing children and young adults (TDs).

**Methods:**

Ambulatory male and female participants with spastic CP, aged 9–24 years, and age- and sex-matched TDs without musculoskeletal disorders were recruited. Participants with CP were measured at baseline, after a three-month control period manifesting normal development, and after the three-month strength, gait, and flexibility training intervention. TDs were measured at baseline and after the control period. They did not attend the intervention. Cardiometabolic risk factors included body weight, body fat percentage, and skeletal muscle mass index assessed with bioimpedance; resting systolic and diastolic blood pressure and aortic pulse wave velocity assessed with a non-invasive oscillometric device; fasting plasma high-density and low-density lipoprotein cholesterol, triglyceride, and glucose levels. Data were analyzed with independent samples t-tests and linear mixed-effects models adjusted for sex and age.

**Results:**

The study involved 18 participants with CP (13 males, 9–22 year, mean 14.2 ± 4.4) and 17 TDs (12 males, 9–22 year, mean 14.6 ± 4.3). At baseline, participants with CP had a 1.0 (95% confidence interval (CI) [-2.0, -0.0]) kg/m^2^ lower skeletal muscle mass index than TDs. During the control period, no statistically significant between-group differences were observed in the change of any outcome. In the CP group, body weight (β = 1.87, 95% CI [1.04, 2.70]), fat percentage (β = 1.22 [0.07, 2.37], and blood glucose (β = 0.19, 95% CI [0.01, 0.37]) increased, while diastolic blood pressure (β=-2.31, 95% CI [-4.55, -0.06]) and pulse wave velocity (β=-0.44, 95% CI [-0.73, -0.16]) decreased. In the TD group, only body weight increased (β = 0.85, 95% CI [0.01, 1.68]) statistically significantly. In the CP group, no changes were observed during the intervention.

**Conclusions:**

Young people with and without CP do not exhibit significant differences in most cardiometabolic risk factors. EXECP intervention may attenuate some adverse development trajectories occurring without the intervention but greater volume and intensity of aerobic exercise may be needed to reduce cardiometabolic risk.

**Trial registration:**

ISRCTN69044459; Registration date 21/04/2017.

**Supplementary Information:**

The online version contains supplementary material available at 10.1186/s13102-024-01006-0.

## Background

Cerebral palsy (CP) is the most common motor disability in childhood, diagnosed in approximately two children per 1000 live births worldwide [1, 2]. CP is described as a permanent, non-progressive neuromotor disorder characterized by abnormal muscle tone, posture, and movement patterns increasing difficulties in activities of daily living and engagement in physical activity and exercise [3]. Therefore, individuals with CP often have physically inactive and sedentary lifestyles, which may lead to a worsening of functional limitations and contribute to chronic disease processes such as elevated cardiometabolic risk [3, 4].

The risk of developing cardiometabolic diseases is high in adults with CP; for example, the prevalence of hypertension, hypercholesterolemia, and type II diabetes are suggested to be 29%, 34%, and 12%, respectively [5]. However, the evidence is somewhat inconclusive as to whether the cardiometabolic disease risk is higher in adults with CP compared to the general population [6]. Moreover, the evidence of whether children and young adults with CP differ from their typically developing peers in cardiometabolic risk factors including adiposity, hypertension, and hyperlipidemia, is scarce [6]. Two recent studies indicate that cardiometabolic risk may be increased already in children and young adults with CP [7, 8]. Batson and colleagues found that children with CP had higher adiposity and fasting serum total cholesterol, LDL cholesterol, non-HDL cholesterol, and glucose levels compared to typically developing children [7], which may predispose them to an elevated risk of cardiometabolic morbidity and mortality in adulthood [9]. In another recent study, Whitney and colleagues found that young adults with CP had a higher risk of developing several cardiometabolic diseases, including hypertension, hyperlipidemia, and myocardial infarction, than individuals without CP [8].

One explanation for the elevated cardiometabolic risk already at a young age may be that children and young adults with CP are less physically active and exercise less than their typically developing peers [10–13]. Similar to the general population [14], high levels of sedentary time and low levels of physical activity have been recognized as risk factors for poor cardiometabolic health in adults with CP [15, 16]. While regular exercise may be an effective tool to improve cardiorespiratory fitness and muscle mass and strength among people with CP [17], research is lacking on whether exercise training effectively reduces cardiometabolic risk, such as blood pressure or lipids, in children and young adults with CP. Strength training is especially effective in improving motor function, muscle mass, and strength [17, 18]. Since muscle tissue is a metabolic organ [4], strength training may have positive effects on cardiometabolic health. In youth with CP, research on the impact of strength training on cardiometabolic risk factors is lacking, but strength training may have small beneficial effects on cardiometabolic risk factors, including adiposity and glucose metabolism, in TD youth [19]. However, because impaired mobility is linked to an increased risk of developing cardiometabolic diseases in people living with CP [20], there is a need to identify feasible and effective interventions to improve mobility and reduce cardiometabolic risk [21]. In our previous study, we found that a three-month, exercise program focusing on intensive and progressive strength training improved muscle strength and motor function, including walking performance, in children and young adults with CP [18], but it is still unclear whether these improvements facilitated improvements also in cardiometabolic health.

Therefore, the purpose of the present study was twofold. First, we investigated if children and young adults with CP had higher cardiometabolic risk compared to typically developing children and young adults. Second, the main aim of the study was to investigate the effects of a three-month exercise intervention including strength, gait, and flexibility training, on cardiometabolic risk factors in children and young adults with CP.

## Method

### Study design and participants

This study reports the secondary outcomes of the EXErcise for Cerebral Palsy (EXECP) study, conducted at the Neuromuscular Research Center and the Center for Interdisciplinary Brain Research, the Faculty of Sport and Health Sciences, University of Jyväskylä, Finland [18, 22]. The EXECP study used a nonconcurrent, multiple baseline design to investigate the effects and mechanisms of a three-month multicomponent exercise intervention in children and young adults with CP. The study protocol was approved by the Ethics Committee of the Central Finland Healthcare District (U8/2017) and was registered prospectively in the International Standard Randomized Controlled Trial Registry (ISRCTN69044459). All participants aged ≥ 18 years and the legal guardians of the underaged participants signed written informed consent. Participants < 18 years signed assent before participation. The detailed study protocol and the results for the main outcome, i.e., six-minute walking distance, have been published previously [18, 22].

Participants for the CP group were recruited from Southern and Central Finland by contacting hospitals, physiotherapy clinics, CP associations, and online CP groups. Eligible participants were males and females aged 9–24 years with confirmed diagnosis of spastic unilateral or bilateral CP, classified as Gross Motor Function Classification System (GMFCS [23]), levels I–III. Exclusion criteria were (a) lower limb surgery and/or pharmacological treatments in the past six months; (b) selective dorsal rhizotomy, i.e., a minimally invasive spinal operation that can permanently reduce leg spasticity and promote independent walking; (c) serial casting on the lower limbs; (d) participation in a resistance training program for the lower limbs in the last six months; (e) inability to sufficiently cooperate in the intervention and testing sessions, i.e., inability to abide by the instructions and perform the tests and training accordingly; (f) inability to stand with both heels touching the floor. Typically developing children and young adults (TDs) were recruited to the control group from local schools and the University of Jyväskylä. Males and females of the age 9–24 years with no musculoskeletal disorders were eligible for the control group.

**Fig. 1:**
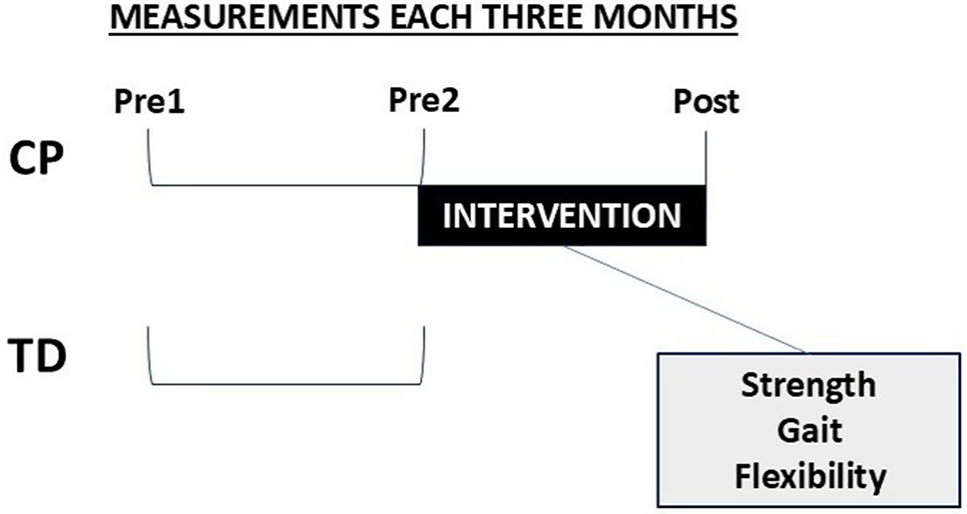
EXErcise for Cerebral Palsy (EXECP) study design. *Abbreviations* *CP* = cerebral palsy group; *TD* = typically developing control group; *Pre1* = the first baseline measurement; *Pre2* = the second baseline measurement; *Post* = post-intervention measurement

The CP group attended three testing sessions: two baseline testing sessions (Pre1 and Pre2) before the intervention started, interspaced by a three-month control period, and a post-intervention testing session immediately after the three-month intervention ended (Post). The length of the control period between Pre1 and Pre2 was equal to the length of the intervention and manifested the potential effects of normal development and activities (e.g., maturation, physiotherapy) during three months. The TD group only attended the two baseline testing sessions (Pre1 and Pre2) interspaced by the three-month control period. They did not participate in the exercise intervention. During the control period, participation in normal physical activities, sports hobbies, and physical therapy, but not in a structured physical training program was allowed for participants in both groups. All laboratory measurements were conducted at the Neuromuscular Research Center and the Center for Interdisciplinary Brain Research. The intervention was performed in various gyms with all necessary equipment, located close to each participant. Participants were recruited between May 29th, 2017, and September 8th, 2020, and data collection for the present analysis ended in December 2020. The study design is presented in Fig. 1.

### Measures

Body weight (kg), body fat percentage (%), and skeletal muscle mass (kg) were assessed with a bioelectrical impedance device (InBody 720, Seoul, South Korea). Body height (cm) was measured with a wall-mounted stadiometer. Skeletal muscle mass index was calculated as skeletal muscle mass divided by height squared (kg/m^2^).

Systolic and diastolic blood pressure (mmHg), and aortic pulse wave velocity (m/s) were measured in a supine position with a non-invasive oscillometric device (Arteriograph, Tensiomed Ltd., Budapest, Hungary) after a 10-minute rest. Measurement was repeated twice, and the mean values of the two measurements were used as the outcomes.

Blood lipids and glucose (mmol/L) were analyzed from venous blood samples drawn after a 12-hour overnight fast. Plasma high-density lipoprotein (HDL) and low-density lipoprotein (LDL) cholesterol, triglycerides, and glucose were measured by enzymatic colorimetric assays (Konelab 20XTi, Thermo, USA).

A continuous cardiometabolic risk score was calculated as a sum of the sex- and age-adjusted standardized residuals from body fat percentage, systolic and diastolic blood pressure, triglycerides, glucose, and the opposite number of HDL cholesterol values [24].

### Intervention

The EXECP intervention, which was composed of strength, flexibility, and gait training, has been described in detail previously [18, 22]. All participants attended two to three weekly supervised, individual training sessions for 12 weeks, and were instructed to do daily gait training at home. Physically active participants, who had a higher number of organized physical activity sessions including physiotherapy and sports hobbies outside the intervention in their weekly schedule, could choose between two and three weekly supervised sessions. More sedentary participants, i.e., those who had fewer organized weekly physical activity sessions outside the intervention, were encouraged to attend three weekly supervised training sessions. The supervisors were physiotherapists or strength and conditioning coaches who had a full understanding of the intervention.

Each supervised training session consisted of 5–10 min of treadmill walking, 60–75 min of strength training, and 0–20 min of flexibility training. Gait training was performed with a portable mechanical treadmill with an adjustable inclination of 6° or 7.3° (Vida XL, Venlo, Netherlands). Participants were instructed to walk at a comfortable pace and could stop to rest at any time. Special attention was paid to gait quality and continuous verbal feedback was given.

Strength training conforming to known training parameter guidelines [17] targeted lower body and trunk muscles. Two alternating strength training protocols, with 5–7 exercises per training session were used. The ten exercises performed were: seated and standing calf raise, seated dorsiflexion, knee extension and flexion, leg press, squat, hip flexion while lying down, trunk extension, and isometric hollow rocks. The focus was on lower limb and trunk muscles since the main outcomes of the EXECP study were the six-minute walking distance and the Gross Motor Function Measure, that is, motor function measures requiring lower limb and trunk muscle strength [18, 22].

The volume of the strength training remained the same across the three months, whereas intensity was increased monthly by adjusting the number of sets and repetitions, movement duration, and rest between sets. In the first month, three sets of eight repetitions maximum (i.e., the ninth repetition could not be executed), with a 3 s concentric and 3 s eccentric movement duration and 60 s of rest were performed. In the second month, the number of sets and repetitions was maintained, but concentric movement duration was reduced to 1 s, and the rest between the sets was increased to 90 s. In the third month, the number of sets was increased to four and the number of repetitions was reduced to six. Concentric movement was done as fast as possible while eccentric movement duration was decreased to 2 s. The duration of rest remained at 90 s. The squat exercise was performed with body weight, with the same movement duration as the other exercises. Difficulty was increased by reducing rest between the sets from 90 to 60 s, adding balance disks below the heels, and using unilateral squats. Trunk extension and hollow rocks were trained isometrically with a progressive increase in duration from 30 to 60 s, and then intensity was increased with additional weights. In all exercises, the load was selected based on the optimal joint angle (i.e., the position where the participant had more strength), and the supervisor assisted in the concentric phase of the movement to ensure full range of the movement excursion.

Flexibility training was only performed for muscle groups that were diagnosed short in the pre-tests [22]. The possible trained muscles were hip flexorsm knee flexors, and hip adductors. Participants with less than 20° of hip extension were diagnosed with short hip flexors; participants with more than 40° of knee flexion in the passive knee extension test were diagnosed with short knee flexors. Hip adductor shortness and the differentiation between one and two-joint hip flexor shortness was inferred from the hip position in the modified Thomas test position [22]. Four sets of 45 s of manual passive-static stretching at the pain threshold were performed for each shortened muscle group.

All participants also received a treadmill to take home and were instructed to walk at least 10 min per day at a comfortable speed. All participants completed twelve weeks of training. Compliance with the supervised training sessions was tracked from training diaries kept by the supervisor. Whenever a participant missed a training session, it was rescheduled for the same or the following week. If necessary, the twelve-week training period was extended by a few days. Compliance with the home-based gait training was tracked from physical activity diaries kept by the participants.

### Statistical methods

Participant characteristics in Pre1 are summarized as means and standard deviations (SD) for both groups separately. Differences between participants in the CP and TD groups in Pre1 were analyzed with Student’s t-test. When inequality of variances was observed in Levene’s test, the Welch test was used instead. Mean differences with 95% confidence intervals (CI) and Hedge’s g effect size estimates are presented.

Linear mixed-effects models were used to investigate changes in cardiometabolic risk factors across the measurement points. First, changes in the outcomes and between-group differences in the changes were investigated during the control period with the full study sample. The main effects of group (CP vs. TD), time (Pre1 vs. Pre2), sex (male vs. female), age, and age^2, and the group-by-time interaction effect were included as fixed effects. Simple effects tests were performed to explore within-group changes. Second, the effects of the intervention on the outcomes were investigated in the CP group only in models including the main effects of time, sex, age, and age^2 as fixed effects. For the time effect, Post was set as the reference category, i.e., post-intervention values were contrasted to both pre-tests. In all analyses, age and age^2 were centered. The participant was included as a random factor to account for the heterogeneity within the study groups. That is, each participant was allowed to have a different y-intercept, but similar slopes.

Normality and homoskedasticity of residuals were assessed by visual inspection using Q-Q plots, residual histograms, and residual-predicted scatterplots. Age^2 was included in the models to improve homoskedasticity after an initial inspection. If clear outliers were observed, residuals were inspected by cluster (i.e., participant). Outliers were excluded from the analysis, if this improved the residual distribution and Akaike’s information criterion value. The results are presented as estimated marginal means (EMM) and unstandardized parameter estimates (β) with standard errors (SE) and 95% CI.

For the first set of linear mixed-effects models assessing the levels of and changes in cardiometabolic risk factors across the control period, intra-class correlation coefficients (ICCs) were calculated to assess the correlation in the outcome in question among observations within the same cluster, i.e., participant. The ICCs were calculated by dividing the random effect variance (σ^2^_i_) by the total variance, i.e. the sum of the random effect variance and the residual variance (σ^2^_ε_).

$$\:\text{ICC}=\frac{{\sigma}^2\text{i}}{{\sigma}^2\text{i}+{\sigma}^2{\epsilon}}$$

In a longitudinal linear-mixed effects model, high ICCs indicate that the repeated observations within clusters are more similar than observations from different clusters and that a high proportion of the overall variation in the outcome is explained by the clusters.

All statistical analyses were performed with jamovi 2.2.5 [25]. Linear mixed-effects models were conducted with the module GAMLj [26]. Participants with missing data were excluded analysis-by-analysis. In linear mixed-effect models, participants with data on at least one measurement time point were included in the analysis. Missing data resulted from participants not consenting to the measurement in question (CP group: body composition *N* = 1 in all measurements; blood pressure *N* = 1 in Post; blood sample *N* = 2 in Pre1 and Pre 2, *N* = 1 in Post; TD: blood sample *N* = 1 in Pre1) and, for pulse wave velocity, unsuccessful measurement (CP: *N* = 1 in Post; TD: *N* = 1 in Pre1). A priori power calculations were performed for the main outcome of the EXECP study, i.e., six-minute walking distance [18, 22].

## Results

### Participant characteristics

Participants of the CP group in the present study were 13 males and five females, aged 9 to 22 years. Fourteen participants were in the GMFCS level I and four in level III. Twelve participants had unilateral and six bilateral CP. Two minor deviations from the predefined exclusion criteria were accepted by the Ethics Committee of Central Finland Health Care District: four participants with medical treatment (epilepsy medication, *n* = 3, and baclofen, *n* = 1) and one participant who had undergone a selective dorsal rhizotomy surgery one year earlier were included in the study. Participants of the TD group were 12 males and 5 females aged 9–22 years.

Participant characteristics measured in Pre1 are presented in Table 1. Participants in the CP group had on average 1.0 kg/m^2^ lower skeletal muscle index and tended to have higher pulse wave velocity than TDs. There were no statistically significant between-group differences in other cardiometabolic risk factors or the overall risk score.

**Table 1:** Participant characteristics at the first baseline measurement by study group, and the differences between children and young adults with cerebral palsy (CP) and typically developing children and young adults (TD)

	CP (*N* = 18)	TD (*N* = 17)		Difference	
	Mean (SD)	Mean (SD)	Mean [95% CI lower, 95% CI upper]	*P* value ^a^	Hedge’s g
Age, yr	14.18 (4.42)	14.57 (4.29)	0.39 [-3.39, 2.60]	0.790	-0.09
Height, cm	158.39 (14.41)	161.06 (16.36)	-2.67 [-13.26, 7.92]	0.611	-0.17
Weight, kg	50.48 (16.51) ^b^	53.10 (17.50)	-2.62 [-14.51, 9.27]	0.657	-0.15
Body fat percentage, %	21.64 (11.04) ^b^	16.36 (7.40)	5.28 [-1.32, 11.88]	0.112	0.55
Skeletal muscle mass index, kg/m^2^	8.08 (1.07) ^b^	9.09 (1.63)	-1.01 [-1.97, -0.05]	0.040	-0.72
Systolic blood pressure, mmHg	113.97 (8.55)	111.53 (10.45)	2.44 [-4.11, 8.99]	0.453	0.25
Diastolic blood pressure, mmHg	62.19 (5.23)	59.85 (5.71)	2.34 [-1.42, 6.10]	0.214	0.42
Pulse wave velocity, m/s	6.33 (0.77)	5.91 (0.43) ^b^	0.42 [-0.02, 0.86]	0.056	0.65
HDL cholesterol, mmol/L	1.53 (0.32) ^c^	1.60 (0.33) ^b^	-0.08 [-0.31, 0.16]	0.515	0.23
LDL cholesterol, mmol/L	2.19 (0.75) ^c^	2.18 (0.61) ^b^	0.01 [-0.48, 0.51]	0.958	0.02
Triglycerides, mmol/L	0.82 (0.31) ^c^	0.77 (0.35) ^b^	0.05 [-0.19, 0.29]	0.674	0.15
Glucose, mmol/L	5.13 (0.44) ^c^	4.98 (0.30) ^b^	0.15 [-0.12, 0.42]	0.269	0.39
Cardiometabolic risk score, total score	0.33 (3.51) ^d^	-1.54 (3.32) ^b^	1.87 [-0.63, 4.38]	0.137	0.54

*Note* *Abbreviations* *SD* standard deviation; *CI* confidence interval; *HDL* high-density lipoprotein; *LDL* low-density lipoprotein

^**a**^P-value from the Student’s t-test, except for body fat percentage and pulse wave velocity from the Welch test

^b^Missing *N* = 1

^c^Missing *N* = 2

^d^Missing *N* = 3

### Changes in cardiometabolic risk factors during the three-month control period

During the three-month control period, weight increased by an average of 1.87 (95% CI [1.04, 2.70]) and 0.85 (95% CI [0.01, 1.68]) kg in the CP and TD groups, respectively (Table 2). In the CP group, this increase was accompanied by a 1.22 (95% CI [0.07, 2.37]) percentage point increase in body fat and a slight increase in skeletal muscle mass index (β = 0.13, 95% CI [-0.01, 0.28]). In addition, diastolic blood pressure (β=-2.31, 95% CI [-4.55, -0.06]) and pulse wave velocity (β=-0.44, 95% CI [-0.73, -0.16]) decreased, while blood glucose increased (β = 0.19, 95% CI [0.01, 0.37]) in the CP group from Pre1 to Pre2. However, no statistically significant between-group differences were observed in the change of any outcome from Pre1 to Pre2. In Pre2, the participants with CP had on average lower skeletal muscle mass index and higher glucose levels than TDs, whereas the initial difference in pulse wave velocity attenuated.

Of note, the ICC values varied notably between the outcomes, indicating that the cluster, i.e. participant, explained a highly varying proportion of the total variance in the outcomes. Within-person variation across the two measurements was very low in all body composition outcomes (ICC: s 0.96–0.99), whereas systolic blood pressure, pulse wave velocity, and triglyceride levels had a relatively high within-person variability (ICC = 0.18–0.42).

**Table 2:** Cardiometabolic risk factors during the three-month control period by study group. The estimated levels of and changes in cardiometabolic risk factors from the first to the second baseline in children and young adults with cerebral palsy (CP) and typically developing children and young adults (TD). Results from the linear mixed-effects models

Group	*N*	Pre1	Pre2	ΔPre2 - Pre1		ΔCP - TD (Pre2)		Group × Time		ICC
		EMM (SE)	EMM (SE)	β (SE)	*p*	β (SE)	*p*	β (SE)	*p*	
Body weight										
CP	17 ^a^	52.37 (3.01)	54.24 (3.01)	1.87 (0.41)	< 0.001	0.15 (4.08)	0.970	1.02 (0.58)	0.086	0.99
TD	17	53.24 (3.07)	54.08 (3.07)	0.85 (0.41)	0.046	Ref.		Ref.		
Body fat percentage										
CP	17 ^a^	23.68 (2.12)	24.90 (2.12)	1.22 (0.56)	0.038	5.57 (2.87)	0.062	0.94 (0.80)	0.247	0.96
TD	17	19.05 (2.16)	19.34 (2.16)	0.28 (0.56)	0.620	Ref.		Ref.		
Skeletal muscle mass index										
CP	17 ^a^	8.03 (0.25)	8.17 (0.25)	0.13 (0.07)	0.071	-0.74 (0.35)	0.042	0.12 (0.10)	0.236	0.96
TD	17	8.89 (0.26)	8.90 (0.26)	0.01 (0.07)	0.875	Ref.		Ref.		
Systolic blood pressure										
CP	18	114.05 (2.22)	112.83 (2.22)	-1.22 (1.26)	0.339	0.91 (3.02)	0.764	-2.28 (1.81)	0.216	0.48
TD	17	110.86 (2.29)	111.91 (2.29)	1.06 (1.30)	0.420	Ref.		Ref.		
Diastolic blood pressure										
CP	18	63.44 (1.14)	61.13 (1.14)	-2.31 (1.10)	0.045	-0.45 (1.56)	0.774	-2.98 (1.58)	0.069	0.82
TD	17	60.91 (1.17)	61.59 (1.17)	0.68 (1.14)	0.556	Ref.		Ref.		
Pulse wave velocity										
CP	18	6.48 (0.13)	6.03 (0.13)	-0.44 (0.14)	0.004	0.11 (0.16)	0.482	-0.35 (0.21)	0.098	0.18
TD	17	6.01 (0.12)	5.92 (0.12)	-0.09 (0.15)	0.534	Ref.		Ref.		
HDL cholesterol										
CP	17 ^a^	1.51 (0.08)	1.60 (0.08)	0.09 (0.06)	0.132	0–02 (0.10)	0.851	0.10 (0.08)	0.189	0.72
TD	17	1.60 (0.08)	1.58 (0.07)	-0.02 (0.05)	0.740	Ref.		Ref.		
LDL cholesterol										
CP	17 ^a^	2.26 (0.16)	2.36 (0.16)	0.10 (0.09)	0.242	0.09 (0.22)	0.694	0.10 (0.12)	0.429	0.86
TD	17	2.26 (0.16)	2.24 (0.16)	0.01 (0.09)	0.939	Ref.		Ref.		
CP	16 ^ab^	0.87 (0.06)	0.89 (0.07)	0.01 (0.07)	0.848	0.09 (0.09)	0.308	0.04 (0.10)	0.700	0.42
TD	17	0.82 (0.06)	0.80 (0.06)	-0.02 (0.07)	0.721	Ref.		Ref.		
Glucose										
CP	17 ^a^	5.15 (0.10)	5.34 (0.10)	0.19 (0.09)	0.044	0.38 (0.13)	0.007	0.21 (0.13)	0.110	0.59
TD	17	4.97 (0.10)	4.95 (0.10)	-0.02 (0.09)	0.839	Ref.		Ref.		
Cardiometabolic risk score										
CP	16 ^c^	0.64 (0.83)	0.43 (0.85)	-0.21 (0.62)	0.737	1.56 (1.13)	0.176	-0.47 (0.84)	0.502	0.74
TD	17	-1.39 (0.82)	-1.13 (0.81)	0.21 (0.57)	0.652	Ref.		Ref.		

*Note* *Abbreviations* *CP* cerebral palsy; *TD* typically developing; *N* number of participants included in the analysis; *Pre1* the first baseline measurement; *Pre2* the second baseline measurement; *ΔPre2 - Pre1* Change from the first to the second baseline measurement; *ΔCP - TD (Pre2)* The estimated mean difference between CP and TD groups in the second baseline measurement; *Group × Time* the difference in change from the first to the second baseline measurement between the groups (CP-TD); *ICC* intra-class correlation coefficient; *EMM* estimated marginal mean; *SE* standard error; *β* unstandardized parameter estimates; *HDL* high-density lipoprotein; *LDL* low-density lipoprotein

^a^One participant was excluded due to missing data in both measurements

^b^One participant was excluded as an extreme outlier based on a visual inspection of the residual Q-Q plot and histogram

^c^Two participants were excluded due to missing data in both measurements

### The impact of the EXECP intervention on cardiometabolic risk in children and young adults with cerebral palsy

In total, the participants with CP performed 24–36 (mean ± SD, 29 ± 4) strength training sessions, which included 32–96 min (67 ± 16 min) of stretching. The total volume of gait training, including supervised training sessions and home training, ranged from 360 to 1984 min (683 ± 352 min). In total, 20 occasions of transient muscle or joint pain were reported by the participants. No severe adverse events were reported.

During the intervention, no statistically significant changes occurred in any cardiometabolic risk factor or the overall risk score in the CP group (Table 3). However, the overall increases of 2.84 (95% CI [1.32, 4.36]) kg in body weight, 2.26 (95% CI [0.69, 3.82]) percentage points in body fat, and 0.19 (95% CI [0.04, 0.35]) kg/m^2^ in skeletal muscle mass index from Pre1 to Post were significant. Post-intervention values of other cardiometabolic risk factors did not differ statistically significantly from Pre1.

**Table 3:** The impact of the three-month EXECP intervention on cardiometabolic risk factors in children and young adults with cerebral palsy. Results from the linear mixed-effects models

			95% Confidence interval		
Outcome	Effect	β	Lower	Upper	*P*
Weight ^**a**^	Intercept	50.21	43.41	57.02	< 0.001
	Time: Pre2 - Post	-0.97	-2.49	0.55	0.216
	Time: Pre1 - Post	-2.84	-4.36	-1.32	< 0.001
	Sex: Female - Male	12.29	-0.88	25.47	0.088
	Age	8.77	2.84	14.70	0.006
	Age^2	-0.22	-0.40	-0.04	0.024
Body fat percentage ^**a**^	Intercept	19.13	14.07	24.19	< 0.001
	Time: Pre2 - Post	-1.03	-2.60	0.53	0.202
	Time: Pre1 - Post	-2.26	-3.82	-0.69	0.007
	Sex: Female - Male	15.88	6.08	25.69	0.007
	Age	0.02	-5.72	5.77	0.993
	Age^2	-0.02	-0.20	0.16	0.850
Skeletal muscle mass index ^**a**^	Intercept	8.36	7.86	8.87	< 0.001
	Time: Pre2 - Post	-0.06	-0.21	0.09	0.447
	Time: Pre1 - Post	-0.19	-0.35	-0.04	0.017
	Sex: Female - Male	-0.17	-1.14	0.80	0.742
	Age	0.70	0.13	1.26	0.020
	Age^2	-0.02	-0.04	-0.00	0.054
Systolic blood pressure	Intercept	114.58	110.15	119.02	< 0.001
	Time: Pre2 - Post	-0.71	-3.85	2.43	0.660
	Time: Pre1 - Post	0.51	-2.63	3.65	0.752
	Sex: Female - Male	-2.86	-10.96	5.24	0.500
	Age	8.08	1.80	14.37	0.021
	Age^2	-0.23	-0.43	-0.04	0.032
Diastolic blood pressure	Intercept	60.40	57.67	63.13	< 0.001
	Time: Pre2 - Post	-1.75	-4.94	1.45	0.292
	Time: Pre1 - Post	0.56	-2.63	3.75	0.733
	Sex: Female - Male	4.61	0.67	8.55	0.038
	Age	2.29	-0.92	5.50	0.183
	Age^2	-0.07	-0.17	0.03	0.179
Pulse wave velocity	Intercept	5.83	5.56	6.11	< 0.001
	Time: Pre2 - Post	-0.15	-0.46	0.17	0.372
	Time: Pre1 - Post	0.30	-0.02	0.61	0.073
	Sex: Female - Male	0.74	0.35	1.14	0.003
	Age	0.37	0.05	0.70	0.039
	Age^2	-0.01	-0.02	-0.00	0.034
HDL cholesterol ^**a**^	Interceot	1.63	1.47	1.79	< 0.001
	Time: Pre2 - Post	-0.02	-0.16	0.12	0.815
	Time: Pre1 - Post	-0.11	-0.25	0.03	0.137
	Sex: Female - Male	0.04	-0.24	0.32	0.803
	Age	-0.27	-0.49	-0.05	0.031
	Age^2	0.01	0.00	0.02	0.028
LDL cholesterol ^**ab**^	Intercept	2.29	1.88	2.69	< 0.001
	Time: Pre2 - Post	-0.12	-0.30	0.07	0.231
	Time: Pre1 - Post	-0.17	-0.36	0.02	0.083
	Sex: Female - Male	0.01	-0.82	0.84	0.984
	Age	-0.16	-0.76	0.43	0.598
	Age^2	0.00	-0.02	0.02	0.680
Triglycerides ^**ab**^	Intercept	0.83	0.68	0.98	< 0.001
	Time: Pre2 - Post	-0.05	-0.22	0.12	0.544
	Time: Pre1 - Post	-0.06	-0.23	0.11	0.495
	Sex: Female - Male	0.17	-0.06	0.39	0.168
	Age	-0.01	-0.19	0.17	0.911
	Age^2	0.00	-0.00	0.01	0.786
Glucose ^**a**^	Intercept	5.29	5.01	5.56	< 0.001
	Time: Pre2 - Post	0.10	-0.10	0.29	0.338
	Time: Pre1 - Post	-0.08	-0.27	0.11	0.429
	Sex: Female - Male	-0.19	-0.68	0.30	0.463
	Age	0.21	-0.17	0.60	0.287
	Age^2	-0.01	-0.02	0.00	0.211
Cardiometabolic risk score ^**c**^	Intercept	0.97	-1.39	3.34	0.431
	Time: Pre2 - Post	-0.71	-2.57	1.15	0.462
	Time: Pre1 - Post	-0.56	-2.38	1.27	0.555
	Sex: Female - Male	0.22	-3.89	4.34	0.917
	Age	1.69	-1.65	5.03	0.338
	Age^2	-0.05	-0.16	0.05	0.320

*Note* *Abbreviations* *EXECP* EXErcise for Cerebral Palsy; *β* unstandardized parameter estimates; *Pre1* the first baseline measurement; *Pre2* the second baseline measurement; *Post* post-intervention measurement; *HDL* high-density lipoprotein; *LDL* low-density lipoprotein

^**a**^One participant was excluded due to missing data in all measurements

^**b**^One participant was excluded as an extreme outlier based on a visual inspection of the residual Q-Q plot and histogram

^c^Two participants were excluded due to missing data in both measurements

## Discussion

We investigated, whether ambulatory children and young adults with CP differed from their typically developing peers in cardiometabolic risk and whether the EXECP intervention lowered this risk in individuals with CP. We found that participants with and without CP did not differ for most cardiometabolic risk markers. In the first baseline measurement, only the skeletal muscle mass index was lower, and arterial pulse wave velocity slightly higher among participants with CP. However, during a three-month control period manifesting normal development and repeatability of the outcome measures, body weight increased in both study groups, while this increase was accompanied by an increase in body fat percentage and blood glucose, and a decrease in diastolic blood pressure and pulse wave velocity only in the CP group. During the three-month individualized and progressive exercise intervention, no statistically significant changes were observed in any cardiometabolic risk factor in participants with CP.

In the first baseline measurement, skeletal muscle mass index was on average lower among participants with CP than TDs, and this difference remained in the second baseline measurement after a three-month control period. During the control period, body weight increased in both study groups, while body fat percentage only increased in the CP group. These findings are in line with most previous studies, although the existing evidence about whether body composition differs between children and young adults with and without CP is scarce and somewhat inconsistent [7, 27–29]. The discrepancies may at least partly be explained by methodological differences. For example, a recent study using bioimpedance, such as the present study, found that children with CP had higher body fat percentage and lower skeletal muscle mass than TDs [28], whereas another study using dual-energy X-ray absorptiometry did not detect any differences in whole-body fat-mass or fat-free mass indices [29]. However, children and young adults with CP may have higher visceral fat although no differences are seen in whole-body fat [7, 29].

In the present study, the increased adiposity was accompanied by increased blood glucose during the control period in the participants with CP. This resulted in higher glucose levels compared to TDs in the second baseline measurement, which is in line with another recent study [7]. The parallel increase in body fat percentage and blood glucose level is reasonable since higher adiposity is associated with higher blood glucose in children and adolescents with and without CP [7, 30]. Both fat and muscle are important metabolic tissues, and increased fat mass along with low skeletal muscle mass may compromise glucose metabolism and increase the severity of functional impairment in CP [4]. Therefore, body composition assessment with a low-cost, easy-to-use, and widely available tool, such as a bioimpedance device, may improve clinical decision-making in identifying children and young adults with CP at increased risk of cardiometabolic diseases. However, measuring waist circumference to assess visceral fat would complement the whole-body fat assessment.

The present study adds to the sparse literature considering blood pressure and lipids in young people with CP. In line with another study conducted among adolescents [31], resting blood pressure did not differ between participants with CP and TDs. However, in contrast to the findings by Martin and colleagues [31], we found that participants with CP tended to have higher pulse wave velocity values compared to TDs. Pulse wave velocity is a sensitive marker of arterial wall stiffness and may thus be an early indicator of cardiometabolic risk [32], and may also contribute to the high prevalence of hypertension observed already in young adulthood among people with CP [8]. It must, however, be noted that both diastolic blood pressure and pulse wave velocity decreased in participants with CP during the three-month control period, and the initial between-group difference in pulse wave velocity was diminished in the second baseline. High within-subject variability in these outcomes indicates that situational factors, such as excitement or mode of transport to the laboratory, may have affected blood pressure assessments.

We did not observe any differences in blood lipids between participants with CP and TDs, which is partly in contrast to two recent studies indicating higher LDL cholesterol values in children with CP [7] and higher prevalence of hyperlipidemia in young adults with CP compared to TDs [8]. One plausible explanation for why we did not find any differences is that the participants with CP in the present study may not have been a representative sample. They agreed to participate in a long and complex exercise intervention and may be healthier and more physically active than the average child or young adult with CP.

The relatively good baseline level of cardiometabolic health may also explain why the three-month exercise intervention did not significantly impact any cardiometabolic risk marker in participants with CP. It is, however, noteworthy that the increase in body weight, body fat percentage, and blood glucose attenuated during the intervention. On the other hand, the beneficial developmental trajectories of diastolic blood pressure and pulse wave velocity were also attenuated. Most of the evidence for the current physical activity recommendations for people with CP is based on the beneficial effects of exercise on cardiorespiratory and musculoskeletal fitness. However, research about the impact of exercise on diverse cardiometabolic risk factors beyond fitness measures is lacking [17]. The training protocol focusing on muscle strength and gait quality, not cardiorespiratory fitness, may have been sufficient to attenuate the unfavorable changes in adiposity and blood glucose, whereas greater volume and intensity of aerobic activity may be needed to reduce blood pressure and blood lipids [33, 34].

This study has several limitations. First, the a priori power calculation was not extended to the outcomes of the present study. Additionally, due to the challenges caused by COVID-19, which broke out while the data collection was ongoing, the target sample size of 24 participants in the CP group was not reached. Thus, the sample size may have been too small to detect differences in cardiometabolic risk factors between participants with CP and TDs on the one hand and changes in the cardiometabolic risk factors on the other. Of note, approximately half of the participants of the CP group attended the study at least partly during the COVID-19 social distancing restrictions, which may have influenced their physical activity levels and eating habits and thus contributed to the observed changes in e.g., body fat and blood glucose. The small sample size also limited the possibility to investigate the effects of, for example, age, sex, or the level of disablement on the outcomes. The age range of the present study is wide, and cardiometabolic risk may develop notably from childhood to young adulthood. Therefore, we adjusted for age, but it was not possible to compare cardiometabolic risk or the effects of the exercise intervention between children, adolescents, and young adults. The small sample size, and within- and between-subject heterogeneity also challenged the robustness of the statistical analyses. Good model fit was not reached for all outcomes. Furthermore, given our small sample size and inherently high between-subject variability in CP, we excluded individuals with specific pharmacological treatments common in people living with CP to reduce this expected variability. Another limitation is that some participants may not have adhered to the 12-hour fasting guidelines before the laboratory visit. Due to these limitations, the results must be interpreted with caution and cannot be generalized to all children and young adults with CP.

This study has several strengths. First, research about cardiometabolic risk, especially biochemical risk markers and the effect of exercise on these risk markers, has thus far been very sparse in children and young adults with CP. Second, although randomized controlled trials are preferred, the multiple-baseline design in which participants acted as their own controls worked well in this heterogeneous target population. Randomization would likely not have resulted in homogenous study groups. The results highlight the need for developing larger-scale studies with a more balanced distribution of participants across genders and GMFCS levels.

## Conclusions

Ambulatory children and young adults with CP and their typically developing peers do not exhibit significant differences in most cardiometabolic risk factors. However, lower skeletal muscle mass index in individuals with CP along with increased adiposity and glucose may predispose them to functional limitations and compromise metabolism. Therefore, intervention strategies such as exercise programs need to be developed to promote healthy body composition and glucose metabolism in this population. Although the EXECP intervention may attenuate some adverse development trajectories occurring without the intervention, it may not effectively reduce cardiometabolic risk. In the future, the impact of exercise interventions with a longer duration and a higher volume and intensity of aerobic exercise on cardiometabolic risk factors should be investigated in individuals CP.

## Electronic supplementary material

Below is the link to the electronic supplementary material.

Supplementary Material 1

## Data Availability

Anonymized datasets generated and analyzed during the current study will be available in the Open Science Framework repository (DOI 10.17605/OSF.IO/4KBJH).

## Abbreviations

CI: Confidence interval
CP: Cerebral palsy
EMM: Estimated marginal mean
EXECP: EXErcise for Cerebral Palsy study
GMFCS: Gross Motor Function Classification System
HDL: High-density lipoprotein
ICC: Intra-class correlation coefficient
LDL: Low-density lipoprotein
Post: The post-intervention test
Pre1: The first baseline test
Pre2: The second baseline test
SD: Standard deviation
SE: Standard error
TD: Typically developing children and adults
